# Protective and Therapeutic Effects of an IL-15:IL-15Rα-Secreting Cell-Based Cancer Vaccine Using a Baculovirus System

**DOI:** 10.3390/cancers13164039

**Published:** 2021-08-11

**Authors:** Van Anh Do-Thi, Hayyoung Lee, Hye Jin Jeong, Jie-Oh Lee, Young Sang Kim

**Affiliations:** 1 Department of Life Sciences, Pohang University of Science and Technology, Pohang 37673, Korea; vananh@postech.ac.kr (V.A.D.-T.); hyejin1597@postech.ac.kr (H.J.J.); 2 Institute of Biotechnology, Chungnam National University, Daejeon 34134, Korea; hlee@cnu.ac.kr; 3 Department of Biochemistry, College of Natural Sciences, Chungnam National University, Daejeon 34134, Korea

**Keywords:** interleukin 15, interleukin 15Rα, B16F10 melanoma, CT-26, cytokine, anticancer

## Abstract

**Simple Summary:**

Interleukin (IL) 15 is a proinflammatory cytokine and is well-known as an efficacious cytokine for cancer immunotherapy. Interestingly, the IL-15:IL-15Rα complex, a self-assembling form of IL-15 and its soluble receptor α (IL-15Rα), has shown greater antitumor activity than IL-15 itself. However, the development of a suitable multigene delivery system is needed for further applications of IL-15:IL-15Rα. Baculovirus, an arthropod-specific virus, is known for its adjuvant effect in cancer therapy. Here, we investigated the potential of the BacMam virus, a modified baculovirus for gene delivery into mammalian cells, as a novel multigene delivery system to generate a cell-based cancer vaccine secreting the self-assembling IL-15:IL-15Rα complex. Vaccination with a BacMam-based IL-15:IL-15Rα cancer vaccine triggered antitumor immune responses in a tumor antigen-specific manner. Our findings indicate that the BacMam system is a safe and effective method to produce protective and therapeutic cancer vaccines.

**Abstract:**

This study reports the use of the BacMam system to deliver and express self-assembling IL-15 and IL-15Rα genes to murine B16F10 melanoma and CT26 colon cancer cells. BacMam-based IL-15 and IL-15Rα were well-expressed and assembled to form the biologically functional IL-15:IL-15Rα complex. Immunization with this IL-15:IL-15Rα cancer vaccine delayed tumor growth in mice by inducing effector memory CD4^+^ and CD8^+^ cells and effector NK cells which are tumor-infiltrating. It caused strong antitumor immune responses of CD8^+^ effector cells in a tumor-antigen specific manner both in vitro and in vivo and significantly attenuated Treg cells which a control virus-infected cancer vaccine could induce. Post-treatment with this cancer vaccine after a live cancer cell injection also prominently delayed the growth of the tumor. Collectively, we demonstrate a vaccine platform consisting of BacMam virus-infected B16F10 or CT26 cancer cells that secrete IL-15:IL-15Rα. This study is the first demonstration of a functionally competent soluble IL-15:IL-15Rα complex-related cancer vaccine using a baculovirus system and advocates that the BacMam system can be used as a secure and rapid method of producing a protective and therapeutic cancer vaccine.

## 1. Introduction

Interleukin (IL) 15 is a member of the cytokine family that all utilize the common gamma-chain (γ_c_) receptor which includes IL-2, IL-4, IL-7, IL-9, and IL-21. IL-15 is functionally the closest cytokine to IL-2 as they share the heterodimeric IL-2/IL-15Rβγ receptor complex to deliver their signals. Unlike IL-2, the specificity and biological activity of IL-15 depends mainly on its private receptor chain IL-15Rα [1,2]. IL-15 is essential for the development, activation, survival, and cytotoxic function of T cells and NK cells. In particular, IL-15 is a critical cytokine that controls memory T cell formation [2,3].

Recently, IL-15 has attracted attention for its promising antitumor effects. Many studies have attempted to exploit the antitumor potential of IL-15 as an immunotherapeutic agent for the treatment of various types of cancer and used a number of different approaches. Among them, the recombinant IL-15:IL-15Rα complex [4,5], IL-15 superagonist [6,7,8], multifunctional IL-15 superagonist [6,9,10,11] and IL-15:IL-15Rα-coated nanoparticles [12,13] have all been tried. Furthermore, combination therapies of IL-15 with nivolumab (anti-PD-1), ipilimumab (anti-CTLA-4), avelumab (anti-PD-L1), alemtuzumab (anti-CD52), obinutuzumab (anti-CD20), and mogamulizumab (anti-CCR4) have all been reported [7,14,15,16,17]. Gene therapy approaches using engineered cells expressing several forms of the IL-15:IL-15Rα complex as cell-based cancer vaccines have also been developed [3,18,19], and we previously showed that a self-assembling IL-15:IL-15Rα complex-expressing cell-based cancer vaccine triggers effective anti-solid tumor responses in mice [3]. However, the traditional method of transfection using lipofectamine for IL-15 and IL-15Rα gene expression hinders its clinical application due to low delivery efficiency, a requirement for the screening process, and lengthy vaccine preparation period. Therefore, a more convenient technique for expressing exogenous IL-15 and IL-15Rα genes in cancer cells is needed.

Several viral vector-based systems including lentivirus and adenovirus have been used to deliver the IL-15 and/or IL-15Rα gene to cell-based cancer vaccines [18,19]. Potential toxicity is the primary concern related to the clinical use of those viral vectors [20,21]. Other weaknesses of the currently available retrovirus, adenovirus, and adeno-associated virus systems are their small DNA cargo size and their limitation as multigene delivery systems [20,22,23]. Compared to other virus-based gene delivery systems, Baculoviridae, a family of insect-specific viruses, have remarkable benefits, among which are low viral cytotoxicity [24,25], very large DNA cargo insertion, and its amenability to multivirus transduction for multimeric protein expression [15,26,27,28,29,30]. Recently, the BacMam vector, a modified vector of baculovirus, was developed as a gene delivery system for mammalian cells [30,31,32,33]. The effective transduction and high-level transgene expression of the baculovirus system are particularly suitable for cancer gene therapy. For example, baculoviruses have been engineered to carry genes of pro-apoptotic factors, toxins, or angiogenesis inhibitors to treat malignancies in xenograft mice [34,35,36]. In particular, a baculovirus-based system may be a powerful gene therapy vehicle for cancer since it works for gene delivery and also plays roles in antitumor responses as an adjuvant [37,38].

In this study, we engineered a BacMam virus (BV) to carry either the self-assembling IL-15 or the IL-15Rα gene and evaluated whether immunization with a BacMam-based IL-15:IL-15Rα complex-expressing cell-based cancer vaccine could provide anticancer protection in mice. We showed that BV can transfer multiple genes to murine cancer cell lines, but transduction efficiency, duration, and level of transgene expression were dependent on cell types, viral dose, and nature of the transgene itself. IL-15 and IL-15Rα delivered by the BV were successfully expressed and assembled to form the IL-15:IL-15Rα complex. Vaccination or treatment with a BacMam-based IL-15:IL-15Rα-expressing cell-based cancer vaccine significantly delayed the growth of B16F10 melanoma and CT26 colon cancer in mice by increasing the population of tumor antigen-specific cytotoxic T cells and stimulating memory T cell formation, as well as by facilitating infiltration of activated T cells and NK cells into tumors.

## 2. Materials and Methods

### 2.1. Animals and Tumor Cell Lines

BALB/C and C57BL/6J mice (female, 6–8-week-old) were purchased from Daehan Biolink (Eumseong, Korea). All animal procedures were approved by the Institutional Animal Care and Use Committee (IACUC) of Chungnam National University (CNU-01056). The murine colon cancer CT26 and the B16F10 melanoma cell lines were cultured in RPMI-1640 (Welgene, Gyeongsan, Korea) supplemented with 10% heat-inactivated fetal bovine serum (FBS) (Sigma-Aldrich, St. Louis, MO, USA), 2 mM L-glutamine, 100 U/mL penicillin, and 100 μg/mL streptomycin (Sigma-Aldrich) in a humidified 5% CO_2_ atmosphere at 37 °C. Sodium butyrate (10 mM, 303410, Sigma-Aldrich) was used to induce baculovirus infection and expression of transgenes. SF9 insect cells were maintained in Sf-900II SFM (Thermo Fisher Scientific, Waltham, MA, USA) supplemented with 5% FBS.

### 2.2. Antibodies and Reagents

The following antibodies were used for FACS staining: APC/Cy7 anti-mouse CD3 (100221, Biolegend, San Diego, CA, USA), FITC anti-mouse CD4 (553729, BD Bioscience, San Jose, CA, USA), FITC anti-mouse CD8 (553031, BD Bioscience), APC anti-mouse CD8 (100711, Biolegend), APC anti-mouse Foxp3 (4331294, Invitrogen, Waltham, MA, USA), APC anti-mouse CD62L (104411, Biolegend), PE anti-mouse CD44 (553134, BD Bioscience), APC anti-mouse CD49b (108909, Biolegend), FITC anti-mouse CD11b (557396, BD Bioscience), H-2K^b^/OVA (SIINFEKL)-Tetramer/PE was kindly provided by Prof. Eui-Cheol Shin (KAIST, Daejeon, Korea).

### 2.3. Generation of OVA-Expressing Cancer Cell Lines

To introduce a CD8^+^ T cell-specific antigen to B16F10 and CT26 cells, a plasmid designed to express membrane-bound ovalbumin peptide (SIINFEKL, OVA) was transfected. Plasmid pCI-neo-OVA was purchased from Addgene (plasmid #25099, Cambridge, MA, USA) [39]. The construct was confirmed by DNA sequencing using OVA-detecting primers (forward primer 5′ ATGGATCAAGCCAGATCAGC 3′ and reverse primer 5′ GCACCAACATGCTCATTGTC 3′). B16F10 or CT26 cells were transfected with the construct using Lipofectamine 2000 (Invitrogen). After 24 h, the cells were selected in a medium containing 0.5 mg/mL G418. To screen for drug-resistant colonies, total RNA was extracted using a Hybrid-R kit (Geneall Biotechnology, Seoul, Korea). Of the total RNA, 1 µg was reverse transcribed using oligo-dT primers and the AccuPower RT premix (Bioneer, Daejeon, Korea) at 42 °C for 1 h. Semi-quantitative PCR was performed using the HSTag premix (Geneall Biotechnology) on a DNA thermal cycler (Bio-Rad, Hercules, CA, USA) using OVA-detecting primers. A B16F10 clone transfected with a mock vector was used as a mock control. E.G7-OVA lymphoma cells, kindly provided by Prof. Yeong-min Park (Konkuk University, Chungju, Korea), were maintained in the complete selection medium containing 0.5 mg/mL G418 and 50 μM β-mercaptoethanol [40].

### 2.4. Recombinant Baculovirus Generation

**Figure 1:**
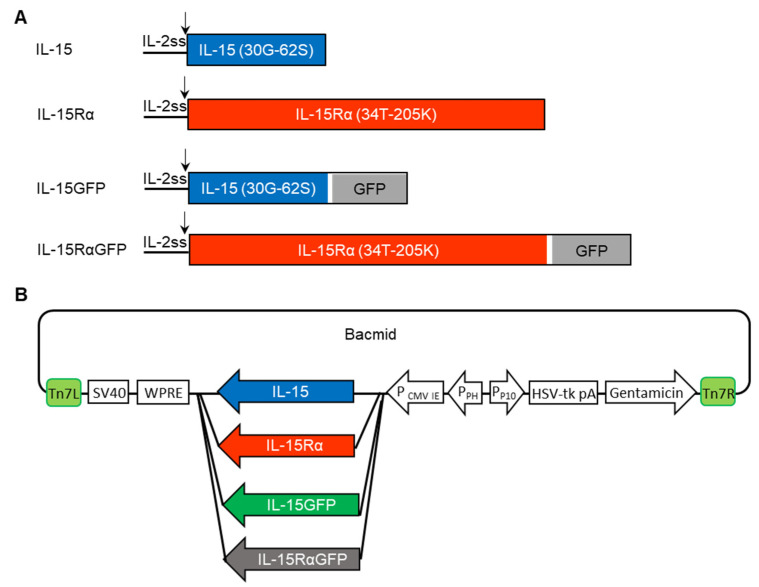
Construction of four different recombinant BacMam viruses (BV). (**A**) Schematic diagram of IL-15, IL-15Rα, IL-15GFP, and IL-15RαGFP proteins. To make the self-assembling IL-15:IL-15Rα complex, the IL-2 signal sequence (IL-2ss, 1M-20S) was used. In the IL-15GFP and IL-15RαGFP fusion proteins, a linker (white) was added. (**B**) Four different bacmids were generated, each of which incorporated a transgene coding sequence (IL-15, IL-15Rα, IL-15GFP, IL-15RαGFP). Some elements are important for high-level expression of the transgene: P_CMV_; CMV promoter, SV40; transcription termination, WPRE; mRNA processing motif. Others are important for baculovirus amplification: P_PH_; promoter of polh, P_P10_; promoter of P10, HSV-tk; terminator, Tn7L and Tn7R; transposon elements, gentamicin; resistance markers.

The replacement of the IL-15 signal peptide with that from IL-2 significantly increased the translation and secretion of IL-15 [41,42,43]. In a previous report, we showed that the IL-2 signal sequence (IL-2ss) facilitated the assembly and secretion of IL-15 and IL-15Rα as a complex when it was used instead of the self-signal sequence of IL-15 or IL-15Rα [3]. In this study, the optimized sequence for expression in murine cell lines of either IL-2ss/IL-15, IL-2ss/IL-15Rα, IL-2ss/IL-15GFP, or IL-2ss/IL-15RαGFP was cloned into the *RsrI*I and *EcoR*I sites of the pEG BacMam vector (Figure 1) [44]. The baculovirus-based pEG BacMam vector containing a CMV promoter was kindly provided by Prof. Eric Gouaux (Howard Hughes Medical Institute, Chevy Chase, MA, USA). The protein sequences of the inserts were shown in Table S1. The generation of recombinant baculoviruses carrying transgenes was generated by homologous recombination between the pEG BacMam vector and the bacmid in DH10Bac *Escherichia coli* cells according to the manufacturer’s instructions (Bac-to-Bac expression system, Invitrogen). After transformation, white colonies were selected and grown in the LB containing 50 μg/mL kanamycin (Sigma-Aldrich), 7 μg/mL gentamicin (Thermo Fisher Scientific), 10 μg/mL tetracycline (Sigma-Aldrich), 40 μg/mL IPTG (Sigma-Aldrich), and 100 μg/mL Bluo-Gal (Gold Biotechology, St. Louis, MO, USA). The final constructions of the four different bacmids are shown in Figure 1B. The recombinant bacmids were isolated using plasmid kits (Qiagen, Hilden, Germany) and transfected into SF9 insect cells using the Cellfectin II reagent (Invitrogen) in the Sf-900II medium (Thermo Fisher Scientific). The virus stock passage (P) 0 was collected and stored at 4 °C. The virus stock was amplified to generate P1, P2, P3 virus stocks in SF-900II containing 5% FBS (Sigma-Aldrich), 1% antibiotic and antimycotic (Invitrogen). The P3 virus stock was used for further experiments. The BacMam viruses were titrated following the rapid method for baculovirus stock titration using GFP reporter expression [45]. The virus was also titrated by the ELISA based on the ability of infected cells to secrete the IL-15:IL-15Rα complex.

### 2.5. In Vitro Viral Delivery

B16F10, CT26, B16F10-OVA, or CT26-OVA cells (3 × 10^5^ cells/60 cm^2^ plate) were transduced with BacMam viruses at various MOI and incubated at 25 °C in 8% CO_2_ in serum-free RPMI. For the delivery of multigenes, the cells were infected with a mixture of two different BacMam viruses at half of the total MOI for each. After incubation for 4 h, the virus inoculum was replaced with complete RPMI containing 10 mM sodium butyrate, and the cells were returned to 37 °C in 8% CO_2_. Twenty-four hours after the viral infection, the cancer cells were harvested and treated with 50 μg/mL mitomycin C (MMC) in PBS for 1 h at 37 °C. After thorough washing of MMC, the inactivated cancer cells were maintained with the complete medium at 37 °C in 5% CO_2_ for an additional time or directly injected into mice.

### 2.6. In Vivo Immunization

B16F10-OVA and CT26-OVA were infected with a mixture of IL-15 BV and IL-15Rα-BV at an MOI of 8 for B16F10-OVA and of 16 for CT26-OVA. Groups of cells were infected with control BV at the same MOI. Twenty-four hours after viral delivery, the cells were harvested and inactivated by treatment with MMC (50 μg/mL) for 1 h. After washing twice with PBS, the inactivated cells were diluted to the final concentration of 3 × 10^5^ or 0.3 × 10^5^ cells/100 μL PBS. One hundred microliters of each condition were used for vaccination. A portion of the virus-infected cells were cultured for an additional 24 h; then, the culture supernatants were collected for the ELISA.

For vaccination, C57BL/6J (*n* = 6 for each group) or BALB/c mice (*n* = 5~6) were injected in the right lower back quadrants twice with a three-day interval (days –31 and –34) with a 3 × 10^5^ MMC-treated cell-based cancer vaccine (no virus, control virus-infected, or IL-15 and IL-15Rα virus-infected cancer cells). For boosting, the mice received the same dosages, two weeks after the first immunization (at days –14 and –17). Fourteen days after the last injection, 5 × 10^5^ live wild-type tumor cells (B16F10-OVA or CT26-OVA cancer cells) were subcutaneously implanted in the left flank. Tumor sizes were measured with a caliper, and tumor volumes were calculated using the following formula: V = (W^2^ × L)/2, where W is the width and L is the length of the tumor [46]. Tumor growth and survival were analyzed. On days 17 and 22 after tumor implantation, blood from tumor-bearing mice was collected by retro-orbital bleeding for further analysis. On day 22 after tumor implantation, the animals were sacrificed, and the spleens and tumor masses were collected.

To examine the therapeutic effect of the BacMam-based OVA cell-based cancer vaccine, 1 × 10^5^ live B16F10-OVA cells were subcutaneously implanted into C57BL/6J mice. At days 3 and 7 after the tumor implantation, 3 × 10^5^ MMC-treated IL-15:IL-15Rα BV- or control BV-infected OVA cell-based cancer vaccine were injected at a nearby position. Tumor growth and tumor weight of those mice were analyzed.

### 2.7. ELISA

ELISAs were used to measure the concentration of the bioactive IL-15:IL-15Rα complex or IL-15 in culture supernatants. In brief, 5 × 10^5^ virus-infected cells were incubated in 2.5 mL of the culture medium in six-well culture plates at 37 °C for 24 h. The IL-15:IL-15Rα complex or IL-15 in the culture supernatants was measured with a mouse IL-15/IL-15R Platinum ELISA (BMS6023, Affymetrix, Santa Clara, CA, USA) or with a mouse IL-15 DuoSet ELISA (DY447-05, R&D system, Minneapolis, MN, USA).

### 2.8. Splenocyte Proliferation Assay

B16F10 cells (5 × 10^5^) were infected with a mixture of IL-15 BV and IL-15Rα BV at the MOI of 10 for each. A group of cells were infected with empty BV (MOI of 20) as controls. Twenty-four hours after the viral infection, the sodium butyrate-containing medium was washed out and replaced by a fresh medium. Culture supernatants from virus-infected cells were collected after additional culturing of 24 h. The splenocytes from normal C57BL/6J mice were plated in the medium containing the culture supernatants (1:10 dilution). After 48 or 72 h, the number of splenocytes was counted to measure proliferation.

### 2.9. Flow Cytometric Analysis

The cells were incubated with an appropriate antibody diluted in the staining buffer (1× PBS containing 0.02% sodium azide and 2% FBS) for 1 h at 4 °C in the dark. After washing off the unbound antibody with a staining buffer, the stained cells were analyzed using a flow cytometer (BD FACSCanto, BD Biosciences). For intracellular staining, the cells were fixed and permeabilized with a fixation and permeabilization solution (554722, BD Biosciences) according to the manufacturer’s instructions. Then, the cells were incubated with an antibody diluted in 0.5% saponin-containing staining buffer for 1 h at 4 °C in the dark. After washing off the unbound antibody, the cells were resuspended in the staining buffer before the FACS analysis. To distinguish dead cells, 10 μg/mL propidium iodide (PI) was added to each sample just prior to the analysis.

### 2.10. In Vitro Stimulation of Splenocytes and the Cytotoxicity Assay

To induce in vitro OVA-specific activation and proliferation, splenocytes from the immunized mice were isolated and stimulated in vitro with the OVA_257–264_ peptide (SIINFEKL, AS-60193, Eurogentech, Seraing, Belgium) following a protocol described in previous reports [47,48]. Briefly, the splenocytes (12 × 10^6^/well) were stimulated in 12-well plates with 2 μg/mL OVA_257–264_ peptide containing RPMI in the presence of 50 μM β-mercaptoethanol and 100 U/mL IL-2 (K0921656, KOMABiotech, Seoul, Korea). After 72 h, the stimulated splenocytes were analyzed using flow cytometry for CD3, CD4, CD8, and Foxp3. For the cytotoxicity assay, 1 × 10^7^ target cancer cells (B16F10-OVA, E.G7-OVA, or mock B16F10) were labeled with 2.5 μM CFSE-containing PBS for 10 min and the remaining CFSE was completely removed. The in vitro stimulated splenocytes with an OVA_257–264_ peptide were used as effector cells. Effector and CFSE-labeled target cells were mixed at different ratios (50:1, 25:1, and 5:1). After 4 h, all the cells were collected and stained with propidium iodide (PI). The percentage of CFSE^+^PI^+^ dead cells was analyzed using flow cytometry (FACSCanto, BD Biosciences).

### 2.11. In Vitro Cell Proliferation Assay

For the analysis of cellular growth rates of live and MMC-treated cancer cells, B16F10-OVA, CT26-OVA, and MMC-inactivated cells (0.5 × 10^4^) were seeded on 96-well plates. The cells were cultured for 96 h and their proliferation was quantitated using the MTT (3-(4,5-dimethylthiazol-2-yl)-2,5-diphenyltetrazolium bromide) assay.

### 2.12. Viral Stability Study

To assess the stability of the BacMam virus during storage (4 °C, in the dark), samples from the same viral stocks were examined for infectious capability at the indicated time points. B16F10-OVA cells (5 × 10^5^ cells/well) were infected with a mixture of IL-15 BV and IL-15Rα BV at the MOI of 10 for each. Culture supernatants were harvested 24 h after the viral infection and the level of IL-15:IL-15Rα was measured using the ELISA.

### 2.13. Statistical Analysis

All the data are presented as the means ± SEM (standard error of mean). GraphPad Prism 7 (GraphPad Software, San Diego, CA, USA) was used for the ANOVA or *t*-test to identify significant differences between the groups, as indicated in figure legends. Survival data were analyzed using the “Kaplan–Meier survival estimates” feature of Origin Pro 8.1 (OriginLab Corporation, Northampton, MA, USA).

## 3. Results

### 3.1. BacMam Viruses Successfully Mediated Multiple Gene Delivery to Murine Cancer Cell Lines

To investigate the BacMam virus (BV) as a gene delivery system for immunotherapy, we used the self-assembling IL-15:IL-15Rα complex as a target cytokine. The self-assembling IL-15:IL-15Rα complex contained two subunit proteins (IL-15 and IL-15Rα) which were guided to intracellular assembly by the IL-2 signal peptide. Then, this signal peptide was cleaved to release the mature protein (Figure 1A). Fusion proteins IL-15GFP and IL-15RαGFP were also constructed to analyze the BV infection and the expression of IL-15, IL-15Rα, or the IL-15:IL-15Rα complex at the single-cell level. The codon-optimized genes for subunits of IL-15 and IL-15Rα were inserted into the bacmid vector by homologous recombination between the pEG BacMam vector and the bacmid in DH10Bac *E. coli* cells (Figure 1B) [44]. Recombinant bacmids were selected and transfected into SF9 insect cells to generate the BV.

**Figure 2:**
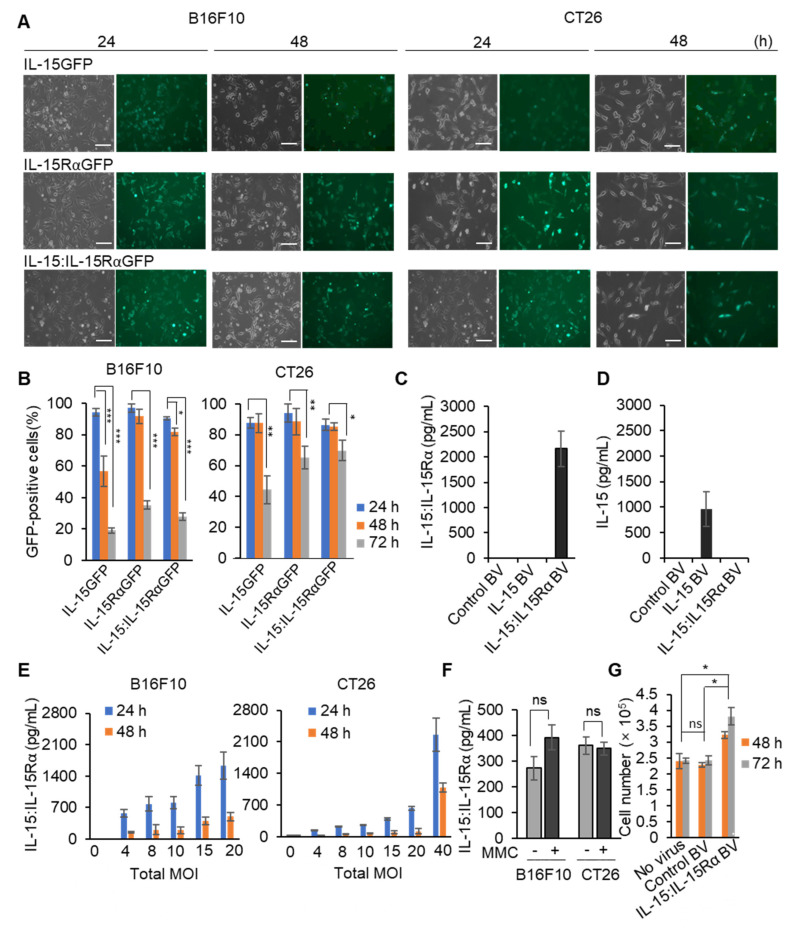
Transduction efficiency in murine cancer cells following infection with BV. (**A**,**B**) B16F10 melanoma or CT26 colon cancer cells were infected with either IL-15GFP BV or IL-15RαGFP BV or both IL-15 BV and IL-15RαGFP BV at the total MOI of 40 and then incubated with a complete medium including sodium butyrate. After 24 h, cells were washed (24-h sample), replaced with a fresh medium without sodium butyrate. After another 24 h and 48 h, the cells were monitored (48-h and 72-h samples, respectively). GFP expression was analyzed under a Nikon fluorescence microscope (scale bar: 100 μm). Left and right figures show the total and GFP-positive cells, respectively (**A**). The infection ratio was estimated by the percentage of GFP-positive cells per the total number of cells. The number of GFP-positive cells was counted using the ImageJ software (**B**). (**C**,**D**) Control BV, IL-15 BV, or the mixture of IL-15 BV and IL-15Rα BV were used to infect 5 × 10^5^ B16F10 cells; 24 h after the viral infection, the concentration of IL-15 or IL-15:IL-15Rα in culture supernatants was quantitated by an IL-15:IL-15Rα complex-specific (**C**) or IL-15-specific (**D**) ELISA kit. (**E**) Cancer cells (3 × 10^5^) were infected with BV at the indicated total MOI (0, 4, 8, 10, 15, 20, 40) in six-well plates. After 24 h, culture supernatants were harvested, replaced using a complete medium, and incubated for an additional 24 h (48-h sample). (**F**) After the 24-h BV infection, the B16F10 or CT26 cells were treated with or without MMC (50 μg/mL) for 1 h and incubated with a complete medium for additional 24 h. The IL-15:IL15Rα expression in the final supernatant was analyzed with an ELISA. (**G**) Naïve mouse splenocytes were treated with culture supernatants from control BV- or IL-15 BV + IL-15Rα BV-infected B16F10 cells for 48 or 72 h. The supernatant from non-infected cells was used as another control (no virus). Cell numbers of the proliferating splenocytes were counted and graphed. Note: * *p* < 0.05, ** *p* < 0.01, *** *p* < 0.001.

Initial studies were conducted to estimate the transduction efficiency of the BV to deliver IL-15GFP, IL-15RαGFP, or both coding genes to B16F10 melanoma and CT26 colon cancer cells. In short, B16F10 melanoma or CT26 colon cancer cells were transduced with either IL-15GFP- or IL-15RαGFP-carrying BV (IL-15GFP BV or IL-15RαGFP BV, respectively) at the MOI of 40. For the codelivery of the IL-15 and IL-15RαGFP genes, the cancer cells were infected with a mixture of IL-15 BV and IL-15RαGFP BV. At the MOI of 40, the GFP signal was detected on almost all the virus-treated cells (more than 80%) at 24 h after the transduction (Figure 2A,B). The GFP expression efficiency was higher for B16F10 cells than for CT26 cells (94% vs. 87%). Since sodium butyrate, a histone deacetylase inhibitor involved in epigenetic regulation of baculovirus gene expression, was administered at the beginning of virus infection and removed after 24 h, the GFP signal gradually waned after 24 h. At 72 h, only 19% of the B16F10 cells or 44% of the CT26 cells showed a detectable IL-15GFP signal, but the expression levels of IL-15RαGFP alone or codelivery of IL-15 + IL-15RαGFP (IL-15:IL-15RαGFP) were higher than that of IL-15GFP in both cancer cells (Figure 2B). For the analysis of the IL-15 BV or IL-15 BV + IL-15Rα BV infection in the absence of GFP, the secreted IL-15 protein or the complex were quantitated by IL-15 monomer-specific or IL-15:IL-15Rα complex-specific ELISA kits (Figure 2C,D). IL-15 BV + IL-15Rα BV-infected B16F10 cells secreted the level of the IL-15 + IL-15Rα complex (IL-15:IL-15Rα) as high as ~2000 pg/mL in 24 h, but the secretion of IL-15 by this transfectant was limited and not at a detectable level. Therefore, IL-15 and IL-15Rα were efficiently assembled to form a biologically functional complex and secreted appropriately into the culture supernatant. The IL-15 BV-infected cells secreted ~1000 pg/mL of IL-15 but not IL-15:IL-15Rα, as expected. The control BV-infected B16F10 secreted neither. The stability of BV during storage was also examined using infectivity-based assays (Figure S1). BV could be maintained at the same activity for more than one year when stored in the dark at 4 °C.

To characterize and optimize the cell-based cancer vaccines secretion, B16F10 or CT26 cancer cells were cotransduced with IL-15 BV + IL-15Rα BV at different MOI, the expression of IL-15:IL-15Rα was quantitated with an ELISA (Figure 2E). The IL-15:IL-15Rα complex expression levels were directly proportional to the MOI. The infected B16F10 cells secreted 3~4-fold more soluble IL-15:IL-15Rα than the infected CT26 cells at the same total MOI. To be used as a cancer vaccine, cancer cells need to be inactivated. Mitomycin C (MMC) is one of the most efficacious agents for inactivation of tumor cells as it inhibits multiple metabolic enzymes [49,50]. MMC treatment completely inhibited in vitro growth of both B16F10 and CT26 cells (Figure S2). To examine the influence of MMC treatment on the duration and levels of IL-15:IL-15Rα expression in MMC-inactivated cells, culture supernatants of the BV-infected cells were tested. As shown in Figure 2F, treatment with MMC did not affect the IL-15:IL-15Rα secretion. To analyze the biological activity of IL-15:IL-15Rα secreted by IL-15 BV + IL-15Rα BV-infected cells, spleen cells from normal C57BL/6J mice were incubated with diluted culture supernatants of the BV-infected cells and control cells for 48 h. The supernatants from the IL-15 BV + IL-15Rα BV-infected cells stimulated spleen cell proliferation 60% more than those from control BV- or no virus-infected cells after 72 h (Figure 2G). This functional assay confirmed that the IL-15 and IL-15Rα coding genes delivered by BV were successfully expressed, and the proteins were self-assembled and functionally competent as a secreted form in the B16F10 and CT26 cancer cells.

### 3.2. BacMam-Based IL-15:IL-15Rα-Secreting Autologous Cancer Cells Triggered Antitumor Protection in Mice

To investigate whether there was a tumor antigen-specific protective effect of the BacMam-based vaccine, we generated B16F10 and CT26 cells expressing an ovalbumin peptide (OVA_257–264_). B16F10-OVA and CT26-OVA cancer cells were infected with IL-15 BV + IL-15Rα BV (termed IL-15:IL-15Rα-B16F10-OVA and IL-15:IL-15Rα-CT26-OVA, respectively). The control BV-infected B16F10-OVA and CT-26-OVA cells were labeled as control-B16F10-OVA and control-CT26-OVA, respectively, from this point. Based on the results shown in Figure 2E, the B16F10-OVA and CT26-OVA cancer cells were coinfected with IL-15 BV + IL-15Rα BV at the MOI of 8 and 16, respectively, and the levels of the IL-15:IL-15Rα complex secreted by each cell line at 0.3 or 3 × 10^5^ cells were measured with an ELISA after 48 h of infection (Figure S3A). IL-15:IL-15Rα-B16F10-OVA produced 25 and 160 pg/mL while IL-15:IL-15Rα-CT26-OVA secreted 11 and 70 pg/mL for 0.3 and 3 × 10^5^ cells, respectively.

Syngeneic C57BL/6J and BALB/C mice were subcutaneously (s.c.) immunized with MMC-treated IL-15:IL-15Rα-B16F10-OVA and IL-15:IL-15Rα-CT26-OVA, respectively, as described in Figure S3B. As noted in Figure 1, the expression level of IL-15:IL-15Rα dropped 2~3 days after the cancer cells were infected with the IL-15 BV and IL-15Rα BV. Therefore, mice were immunized twice at an interval of 3 days to achieve the steady-state level of IL-15:IL-15Rα for vaccination immune responses. Two weeks after immunization, the mice were challenged with live B16F10-OVA and CT26-OVA cells. The mice immunized with 3 × 10^5^ IL-15:IL-15Rα-B16F10-OVA or IL-15:IL-15Rα-CT26-OVA per mouse, a dose which secreted IL-15:IL-15Rα at 70~160 pg/mL after 48 h, showed significantly delayed cancer cell growth compared with the groups immunized with no virus-infected or control BV-infected cancer cell groups (Figure S3D,F). The mice immunized with 3 × 10^5^ IL-15:IL-15Rα-B16F10-OVA or IL-15:IL-15Rα-CT26-OVA showed similar body weight changes to the no virus-infected cancer cells, indicating the tolerability of BV-based cancer cell immunization (Figure S3G,H). At the lower dose of 0.3 × 10^5^ cells/mouse which secreted IL-15:IL-15Rα in the range of 10–25 pg/mL, the immunization did not inhibit tumor growth effectively (Figure S3C,E). Based on these studies, the cell concentration that produced 160 pg/mL for IL-15:IL-15Rα-B16F10-OVA and 70 pg/mL for IL-15:IL-15Rα-CT26-OVA after 48 h was used for further experiments (Figure S3A).

**Figure 3:**
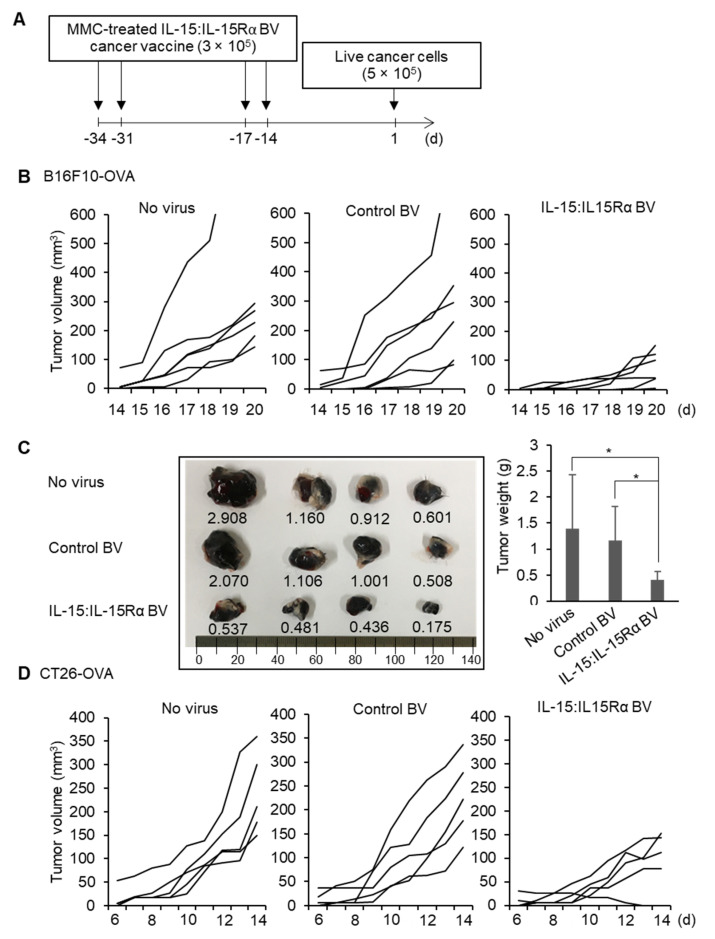
Protective effect of the BacMam-based IL-15:IL-15Rα cancer vaccine against B16F10-OVA melanoma and CT26-OVA colon cancer. (**A**) Optimal vaccination schedule; BacMam-based cell-based cancer vaccines were prepared by infecting B16F10-OVA or CT26-OVA cells with IL-15 BV + IL-15Rα BV at the total MOI of 8 or 16. Then, mice were subcutaneously vaccinated with IL-15:IL-15Rα-B16F10-OVA or IL-15:IL-15Rα-CT26-OVA (3 × 10^5^ cells/mouse) in the right flank after MMC inactivation of the cells on the indicated days. Two weeks after the last immunization, the immunized mice were subcutaneously challenged with either B16F10-OVA or CT26-OVA cancer cells (5 × 10^5^ cells/mouse) in the left flank. (**B**,**C**) Protective effect of the BacMam-based OVA cell-based cancer vaccine against B16F10-OVA melanoma in C57BL/6J mice (*n* = 6). Tumor growth curves of B16F10-OVA cells in the C57BL/6J mice vaccinated with IL-15:IL-15Rα-B16F10-OVA (IL-15:IL-15Rα BV), control-B16F10-OVA (control BV), or non-infected B16F10-OVA cells (no virus) (**B**). Typical tumor samples (left) and the average tumor weights at day 22 (right) (**C**). (**D**) Tumor growth curves of CT26-OVA cells in the BALB/c mice vaccinated with IL-15:IL-15Rα-CT26-OVA (IL-15:IL-15Rα BV), control-CT26-OVA cells (control BV), and non-infected CT26-OVA cells (no virus) (*n* = 5). Note: * *p* < 0.05.

Next, we boosted immunizations with IL-15:IL-15Rα-B16F10-OVA with the same doses 2 weeks after the priming injection to analyze whether the boosting immunization would protect against future exposure to live cancer cell injection in mice (Figure 3A). C57BL/6J mice (*n* = 6) were s.c. immunized with 3 × 10^5^ MMC-inactivated IL-15:IL-15Rα-B16F10-OVA at days –34, –31, –17, and –14 on the left flank. The other groups were injected with either control-B16F10-OVA or no virus-infected cancer cells as controls. Two weeks after the last immunization, all the immunized mice were s.c. challenged with 5 × 10^5^ live B16F10-OVA tumor cells on the right flank. Immunization with IL-15:IL-15Rα-B16F10-OVA significantly delayed B16F10 tumor growth compared to the mice immunized with no virus-infected B16F10-OVA and control-B16F10-OVA (Figure 3B). Tumor weights at day 22 of the mice immunized with IL-15:IL-15Rα-B16F10-OVA were smaller than those of the controls 3–3.5-fold (Figure 3C). A similar experiment using IL-15:IL-15Rα-CT26-OVA also showed significantly reduced cancer growth compared to the control groups (Figure 3D).

### 3.3. Immunization with IL-15:IL-15Rα-B16F10-OVA Induced a Robust Antitumor Response Depending on Tumor Antigen-Specific CD8^+^ T Cells

**Figure 4:**
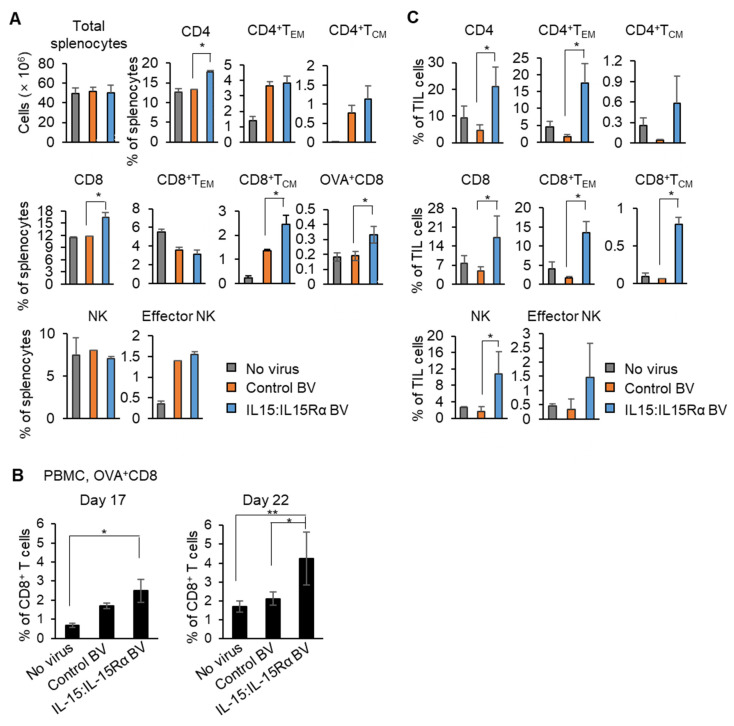
The BacMam-based IL-15:IL-15Rα-B16F10-OVA vaccine induced strong tumor antigen-specific T cell and NK cell responses in mice. (**A**–**C**) The mice were immunized with IL-15:IL-15Rα-B16F10-OVA (IL-15:IL-15Rα BV), control-B16F10-OVA (control BV), or no virus-infected B16F10-OVA (no virus), as shown in Figure 3A (*n* = 4~5) and challenged with live B16F10-OVA cells. (**A**) Splenocytes were collected at day 22 and stained to analyze for the percentage of effector memory T cells (T_EM_, CD44^+^CD62L^–^), central memory T cells (T_CM_, CD44^+^CD62^+^), OVA-specific CD8^+^ T cells (H-2K^b^/OVA (SIINFEKL)-Tetramer^+^CD3^+^CD8^+^), and the NK subset (total NK cells; CD49b^+^, effector NK; CD49b^+^CD11b^+^). (**B**) PBMC were harvested on day 17 and 22 and stained to analyze OVA-specific CD8^+^ T cells (H-2K^b^/OVA (SIINFEKL)-Tetramer^+^CD3^+^CD8^+^). (**C**) The TILs from the vaccinated mice were collected at day 22 and stained to analyze effector memory and central memory T cells and NK subsets. Note: * *p* < 0.05, ** *p* < 0.01, OVA^+^CD8; OVA-specific CD8^+^ T cells.

In the same experiment as shown in Figure 3B, the tumor-bearing mice were sacrificed to collect spleens at day 22 after challenge with B16F10-OVA cells. Proportions of T and NK cells were analyzed with FACS. FACS gating used for the analysis of splenocytes is shown in Figure S4. Although there was no significant change in the total number of splenocytes, CD4^+^ or CD8^+^ splenic T cells were increased 1.4-fold in the mice immunized with IL-15:IL-15Rα-B16F10-OVA compared with those of the control-B16F10-OVA group (Figure 4A). Furthermore, the percentages of the central memory, CD44^+^CD62L^+^, CD8^+^ subset increased 1.9-fold, and OVA-specific CD8^+^ T cells increased 1.7-fold in the experiment using the H-2K^b^/OVA (SIINFEKL)-Tetramer. NK cell percentages in the mice immunized with IL-15:IL-15Rα-B16F10-OVA was not changed in the spleen. To determine whether tumor antigen-specific CD8^+^ T cells are present in the blood of immunized mice, peripheral blood mononuclear cells (PBMC) from the blood of tumor-bearing mice was collected at days 17 and 22 after B16F10-OVA cell challenge and analyzed for the frequency of OVA-specific CD8^+^ T cells. FACS gating used for analysis of the OVA-specific CD8^+^ T subset is shown in Figure S5. The percentages of OVA^+^CD8^+^ T cells among the total PBMC of the IL-15:IL-15Rα-B16F10-OVA-immunized mice were 1.5- and 2-fold higher than in the control-B16F10-OVA group at days 17 and 22, respectively (Figure 4B). The fold changes were more significant when compared with those of the mice immunized with no virus-infected B16F10-OVA cells (3.6- and 2.5-fold increase at days 17 and 22, respectively).

An essential property of a successful cancer vaccine is that it induces infiltration of T cells into the tumor region. Therefore, the characteristics of tumor-infiltrating lymphocytes (TILs) were evaluated in the immunized mice sacrificed on day 22 after the live cancer cell challenge. FACS gating used in the analysis is shown in Figure S6. The total CD4^+^ and CD8^+^ TILs from the mice immunized with IL-15:IL-15Rα-B16F10-OVA increased 4.5- or 3.6-fold, respectively, compared to the controls (Figure 4C). Notably, the percentages of both effector memory and central memory CD8^+^ T cells increased significantly, 8–12-fold, in TILs from the mice immunized with IL-15:IL-15Rα-B16F10-OVA. The percentages of both types of memory cells in the CD4^+^ subset also increased 11–16-fold. Interestingly, the NK cell portion was also elevated sevenfold in the TIL population of the mice immunized with IL-15:IL-15Rα-B16F10-OVA. Collectively, the BV-based IL-15:IL-15Rα cell-based cancer vaccine induced potent antitumor responses in both the spleen and the circulatory system through a tumor antigen-specific CD8^+^ T cell-dependent mechanism. Further, this antitumor response resulted in a more dramatic improvement of CD4^+^, CD8^+^, and NK cell infiltration into the tumor.

### 3.4. Immunizing with the IL-15:IL-15Rα-B16F10-OVA Vaccine Specifically Enhanced Cytotoxic T Cell Activity against B16F10-OVA Cells

**Figure 5:**
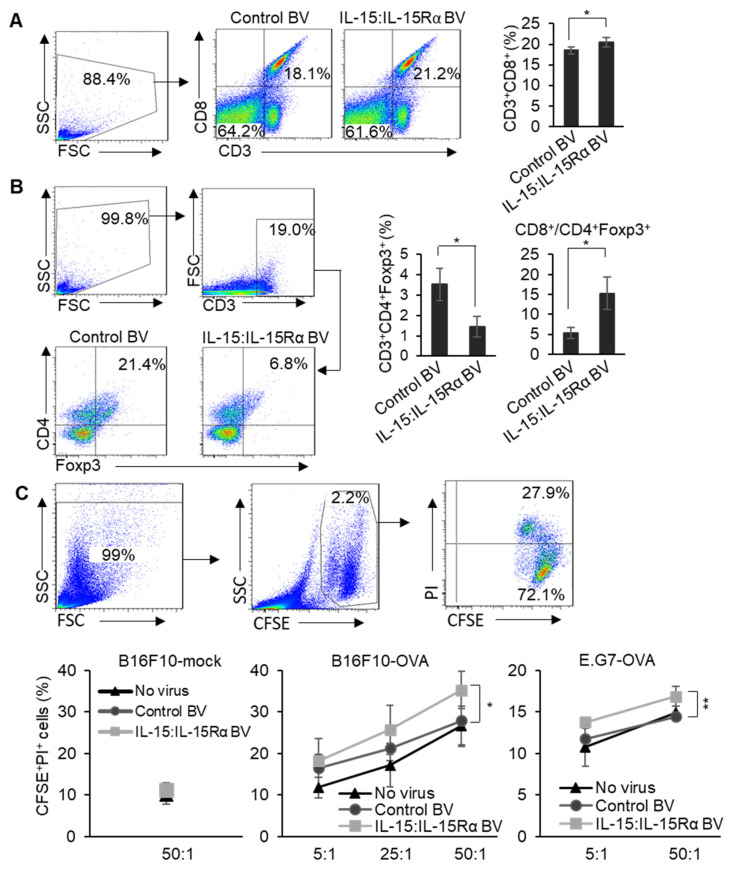
The IL-15:IL-15Rα-B16F10-OVA vaccine enhanced cytotoxicity of OVA-specific CD8^+^ T cells. Mice were immunized as shown in Figure 3A (*n* = 4~5). On day 22 after challenging with live B16F10-OVA cells, splenocytes from the tumor-bearing mice were collected and stimulated with OVA peptides, SIINFEKL, and IL-2 (20 U/mL) for 72 h in vitro. (**A**) The stimulated splenocytes were analyzed for CD3^+^CD8^+^. (**B**) CD3^+^CD4^+^Foxp3^+^ (Treg cells) and the ratio of CD3^+^CD8^+^ to CD3^+^CD4^+^Foxp3^+^ were analyzed. (**C**) The OVA peptide-stimulated splenocytes and CFSE-labeled target cells (B16F10-mock, B16F10-OVA, E.G7-OVA) were mixed at the ratios of 5:1, 25:1, and 50:1. The percentages of CFSE^+^PI^+^ cells (dead cells) were analyzed with the flow cytometric analysis. Note: * *p* < 0.05, ** *p* < 0.01, B16F10-mock; mock-transfected B16F10.

To further evaluate the effect of the IL-15:IL-15Rα-B16F10-OVA vaccine on tumor-specific cytotoxic T cell activity, C57BL/6J mice (*n* = 5) were vaccinated as shown in Figure 3A. The splenocytes derived from tumor-bearing mice then underwent antigenic stimulation with treatment with an OVA peptide in the presence of IL-2 for 72 h in vitro. Following this, the OVA peptide-stimulated splenocytes were analyzed for cytotoxic T cells (Tc) or regulatory T cells (Treg). The percentage of CD8^+^ Tc cells in the splenocytes of the mice immunized with IL-15:IL-15Rα-B16F10-OVA was increased 1.2-fold compared to the control-B16F10-OVA immunized group (Figure 5A). However, a significant reduction (2.4-fold) in the portion of Foxp3^+^ Treg cells was observed, and the CD8^+^/CD4^+^Foxp3^+^ ratio increased three times in the group immunized with the IL-15:IL-15Rα-B16F10-OVA vaccine (Figure 5B). The CD8^+^/CD4^+^Foxp3^+^ ratio is known to be a marker that correlates with prolonged cancer patient survival.

Next, to evaluate the efficacy of antigen-specific effector and memory CD8^+^ T cells, we tested an in vitro model, in which only OVA-specific T cells were expanded by treatment with an OVA peptide, and it was hypothesized that only the expanded and activated OVA-specific CD8^+^ T cells would then be able to exert specific cytotoxic activity against OVA-expressing target cancer cells [47,48]. Therefore, we analyzed the cytotoxic activity of OVA peptide-stimulated splenocytes obtained from the mice immunized with IL-15:IL-15Rα-B16F10-OVA, control-B16F10-OVA cells, or the “no virus group” against two OVA-expressing cancer cell lines, B16F10-OVA or E.G7-OVA lymphoma cells. The negative control was B16F10-mock, a mock-transfected B16F10 cell line which is OVA-negative. Against B16F10-OVA or E.G7-OVA cells, the OVA peptide-stimulated splenocytes from all the groups showed enhanced cytotoxicity which correlated with the ratio of splenocytes vs. the target cancer cells (Figure 5C). However, the splenocytes derived from the mice immunized with the IL-15:IL-15Rα-B16F10-OVA vaccine showed a significantly enhanced OVA-specific cytotoxicity against both B16F10-OVA and E.G7-OVA cancer cells compared with those from the groups infected with control BV or no virus at all the ratios. The cytotoxic potential of splenocytes from all of the OVA peptide-stimulated groups against B16F10-mock cells was similar and close to the basal level.

### 3.5. The Therapeutic Effect of Post-Treatment with IL-15:IL-15Rα-B16F10-OVA

**Figure 6:**
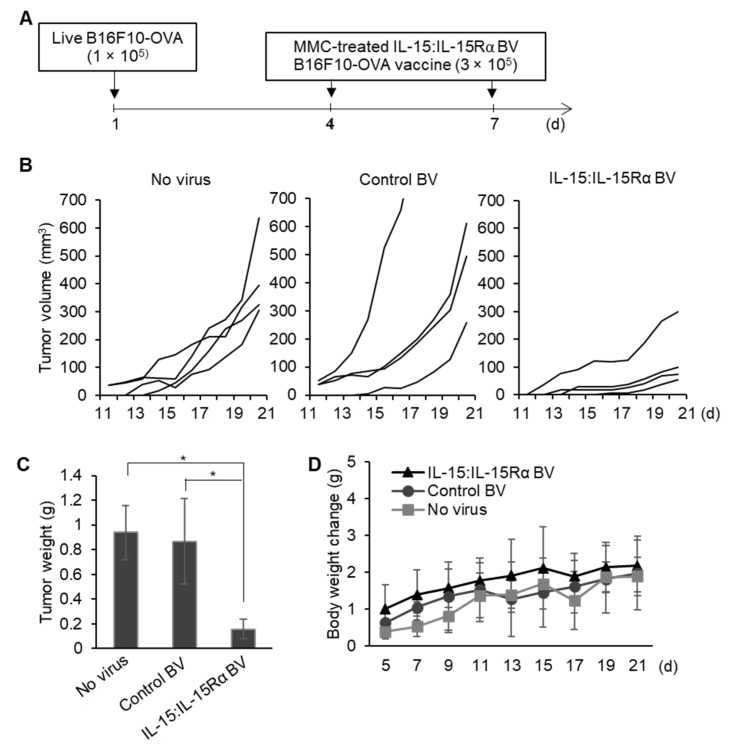
Therapeutic effect of the BacMam-based B16F10-OVA cell-based cancer vaccine. (**A**) Schedule of tumor cell injections. C57BL/6J mice were implanted with B16F10-OVA cells (1 × 10^5^ cells/mouse) subcutaneously in the right flank (*n* = 4 for each group). Four and seven days after the tumor implantation, the tumor-bearing mice were injected with MMC-inactivated IL-15:IL-15Rα-B16F10-OVA (IL-15:IL-15Rα BV), control-B16F10-OVA (control BV), or no virus-infected B16F10-OVA cells (no virus) (3 × 10^5^ cells/mouse) at nearby sites. (**B**–**D**) Tumor growth curves (**B**), tumor weights at day 21 after the tumor implantation (**C**), and changes in the body weight (**D**); * *p* < 0.05.

Finally, we examined whether treatment with the IL-15:IL-15Rα-B16F10-OVA vaccine could delay autologous cancer cell growth in mice. C57BL/6J mice were s.c. implanted with 1 × 10^5^ live B16F10 cells. On days 4 and 7 after tumor implantation, the mice were treated with MMC-inactivated IL-15:IL-15Rα-B16F10-OVA by s.c. injection at nearby sites (Figure 6A). Other groups were treated with MMC-inactivated control-B16F10-OVA or non-infected cells as controls. Treatment with the MMC-inactivated IL-15:IL-15Rα-B16F10-OVA vaccine exerted a marked delaying effect on the growth of B16F10 cells (Figure 6B). The tumor weights from the mice treated with the IL-15:IL-15Rα-B16F10-OVA vaccine were a fifth of those from the control groups after 21 days of tumor implantation (Figure 6C). No major difference in body weights were observed between the three experimental groups, indicating the tolerability of BV-based cancer cell treatment (Figure 6D).

## 4. Discussion

We previously showed that IL-15:IL15Rα-secreting cell-based cancer vaccine surpassed those expressing IL-15 alone for eliminating tumors, preventing tumor recurrence and enhancing overall survival of tumor-bearing mice in CT26 colon cancer cells [3]. However, a safer and more efficient multigene delivery system to produce vaccines is required for clinical applications. In this study, we introduced a new gene delivery strategy using BV to transfer and express the IL-15 and IL-15Rα genes in murine cancer cells as well as to facilitate the intracellular assembly of proteins encoded by those genes. Using this method, we prepared a BacMam-based IL-15:IL-15Rα-secreting cell-based cancer vaccine which triggered a robust antitumor immune response in mice and, of particular note, induced cytotoxic CD8^+^ T cell activation.

To develop a suitable viral-based multigene delivery system for a cell-based vaccine that would secrete the IL-15:IL-15Rα complex, we constructed four different BacMam viruses including IL-15 BV, IL-15Rα BV, IL-15GFP BV, and IL-15RαGFP BV. After infection with a BV, both the IL-15 and IL-15Rα proteins were well-expressed and could dimerize to form the biologically functional IL-15:IL-15Rα complex in either B16F10 or CT26 cancer cells. The complex, upon dimerization, was also secreted in significant amounts by the cancer cells. Worth noting is also the fact that individual transgene production can be easily regulated by adjusting viral doses. In terms of the expression level, the BacMam-based IL-15:IL-15Rα CT26 vaccine, at the total MOI of 40, secreted the protein complex approximately 1000-fold stronger than the previous vaccine made using conventional transfection methods with lipofectamine [3]. In comparison with the adenovirus system, IL-15 BV-infected B16F10 at the MOI of 10 expressed more IL-15 than IL-15-adenovirus-infected TRAMP-C2 or TS/A cells at the MOI of 100 [18]. However, the BacMam-based IL-15:IL-15Rα secretion was transient and dependent on sodium butyrate treatment. The cellular morphology, expression strength, and percentages of GFP-expressing cells indicated that transgene expression and its duration after the infection depended not only on the viral dose, but also on the cell type and the nature of the transgene itself. Since IL-15 has several functions in common with IL-2, the potential toxicity of high dosages of IL-15 is an important safety consideration. Increased IL-15 has been found in many autoimmune diseases, and the transgenic overexpression of IL-15 leads to leukemia in mice [16]. Administration of exogenous IL-15 might cause systemic toxicity in humans and mice by hyperproliferation of activated NK cells and escalation of plasma IFN-γ [51,52,53]. Therefore, we determined the working doses of the BacMam-based cancer vaccine for mice by conducting a dose–response study and the cell concentration that produced the optimum level of IL-15:IL-15Rα was selected for further experiments. BV was very stable under standard storage conditions based on the observation that the infection capability of the BV stock did not change after 12 months. Collectively, BV is a powerful tool for co-expressing multiple proteins in the cancer cell models we tested.

Immunization with BacMam-based IL-15:IL-15Rα-secreting cancer cells successfully raised memory immune responses against solid tumors in mice. We showed that the MMC-treated IL-15:IL-15Rα-B16F10-OVA vaccine inhibited the tumor growth of B16F10-OVA by enhancing splenic central memory CD8^+^ T cells, tumor-infiltrating NK cells, and tumor-infiltrating effector memory CD4^+^ and CD8^+^ T cells. In addition, splenocytes and PBMC from the mice immunized with the IL-15:IL-15Rα-B16F10-OVA vaccine showed increases in the OVA-specific CD8^+^ T subset. It was also shown that splenocytes from the mice injected with the IL-15:IL-15Rα-B16F10-OVA vaccine enhanced tumor antigen (OVA)-specific cytotoxicity against both B16F10-OVA and E.G7-OVA when they were stimulated with an OVA peptide in vitro. Although E.G7-OVA and B16F10-OVA cells may express different kinds of tumor antigens, they share the H-2K^b^/OVA epitope, SIINFEKL. Therefore, it is possible to conclude that higher OVA-specific memory T cells were developed by the IL-15:IL-15Rα-B16F10-OVA vaccine. Interestingly, while vaccination with control BV-infected cells caused the accumulation of Treg cells in response to OVA-specific stimulation in vitro, immunization with IL-15 BV + IL-15Rα BV-infected cells (such as IL-15:IL-15Rα-B16F10-OVA) lowered the percentages of the Treg, which is consistent with the enhanced function of antitumor cytotoxic T cells functioning in an OVA-specific manner. Finally, we also showed therapeutic effects on B16F10-OVA melanoma by treatment with the MMC-treated IL-15:IL-15Rα-B16F10-OVA vaccine in mice. Surprisingly, the analysis of antitumor immune responses induced by vaccination with control BV-infected B16F10-OVA cells often revealed higher responses than “no virus” B16F10-OVA cells. This could occur due to BV itself working as an adjuvant and may be evidence for an additional immune-stimulating effect. In fact, the adjuvant effect of BV against cancer has previously been reported. For example, immunization with BV combined with the tumor cell lysate effectively elicited activation of dendritic cells loaded with the tumor lysate antigen. In turn, the activated dendritic cells induced strong antitumor immune responses to constrain tumor growth [37]. BV was also reported to trigger innate immune responses in mice by inducing the secretion of IL-12, IL-6, TNF-α, and type I IFN by way of TLR9- and TLR3-dependent pathways [38].

Both lentivirus and adenovirus systems have been used to make IL-15:IL-15Rα cell-based cancer vaccines for immunotherapy [18,19]. Co-expression of IL-15 and membrane-associated IL-15Rα by an adenovirus system exerted stronger antitumor effects than IL-15 alone against murine breast and prostate cancer [18]. Berger et al. also demonstrated that the expression of the IL-15-sushiIL-15R fusion protein by a lentivirus system extended survival of mice in a leukemia model. However, IL-15-sushiIL-15R cancer immunotherapy showed lower antitumor efficiency than IL-15 alone [19]. There may be several reasons why the two reports produced different results. One reason could relate to the different types of tumor models, i.e., solid cancer vs. hematopoietic malignancy. The relative concentration of the biologically functional IL-15 and IL-15:IL-15R and their toxicity could be another.

Whole tumor cell vaccines have been studied for several decades with one prominent example being Melacine, a melanoma vaccine, that showed remarkable antitumor activities in phase III of a clinical trial for cancer treatment and was approved to treat malignant melanoma in Canada [54,55,56]. Despite this success, the clinical efficacy of many other tumor cell vaccines remains modest. In addition, further applications of whole tumor cell therapy are greatly hampered by the limitation of patient materials [57] and difficulties in vaccine preparation [55]. Over the last several years, the clinical potential of immune-based therapies in cancer treatment, including T cell checkpoint inhibitors, chimeric antigen receptor-based therapies, and dendritic cell-based cancer vaccines, has attracted much attention from investigators and investors [58,59]. The success of these immunotherapies is likely to have been the stimulus for the recent development of new cancer vaccines utilizing the concept of combination treatments [54,56,57,60,61]. Further research on BacMam-based tumor cell vaccines may attract even more interest in the clinical development of anticancer agents as a powerful tool for genetic therapies and cancer immunotherapeutics.

## 5. Conclusions

For the first time, we report that BacMam virus can be used as a powerful tool for making autologous cell-based cancer vaccines. BacMam-based IL-15 and its soluble IL-15Rα were well-expressed and intracellularly assembled to form a biologically functional IL-15:IL-15Rα complex for secretion. Immunization with the BacMam-based IL-15:IL-15Rα cancer vaccine significantly delayed tumor growth by stimulating robust antitumor immune responses in tumor antigen-specific CD8^+^ T cells and NK cells while simultaneously attenuating Foxp3^+^ Treg cells.

## Data Availability

The data presented in this study are available on request from the corresponding author. The data are not publicly available due to security issues.

## Supplementary Materials

The following are available online at https://www.mdpi.com/article/10.3390/cancers13164039/s1, Table S1. Amino acid sequences of murine IL-15 and IL-15R α and their fusion proteins, Figure S1. BacMam virus stability, Figure S2. Proliferation assay of MMC-treated tumor cells, Figure S3. Determination of effective doses of the BacMam-based IL-15:IL-15Rα cancer vaccine, Figure S4. The BacMam-based IL-15:IL-15Rα-B16F10-OVA vaccine increased the tumor antigen-specific T cell and NK cell subsets in the spleen, Figure S5. The BacMam-based IL-15:IL-15Rα-B16F10-OVA vaccine increased the OVA-specific CD8 T cell subset in PBMC, Figure S6. The BacMam-based IL-15:IL-15Rα-B16F10-OVA vaccine increased the T cell and NK cell subsets in tumor-infiltrating lymphocytes (TILs).
