# Supplementary material for: Protective and Therapeutic Effects of an IL-15:IL-15Rα-Secreting Cell-Based Cancer Vaccine Using a Baculovirus System
Source: Cancers (Basel). 2021 Aug 11;13(16):4039. doi: 10.3390/cancers13164039 (PMC8394727; doi:10.3390/cancers13164039)
Supplement: Supplementary file 1 [file cancers-13-04039-s001.zip › cancers-1285019-supplementary.pdf]

Supplementary Table

Table S1 Amino acid sequences of murine IL-15 and IL-15Rα and their fusion proteins

Proteins	Amino acid sequences
IL-15	MYSMQLASCVTLTLVLLVNSGIHVFILGCVSVGLPKTEANWIDVRYD<br>LEKIESLIQSIHIDTTLYTDSDFHPSCKVTAMNCFLLELQVILHEYSNM<br>TLNETVRNVLYLANSTLSSNKNVAESGCKECEELEEKTFTEFLQSFI<br>RIVQMFINTS
IL-15-GFP	MYSMQLASCVTLTLVLLVNSGIHVFILGCVSVGLPKTEANWIDVRYD<br>LEKIESLIQSIHIDTTLYTDSDFHPSCKVTAMNCFLLELQVILHEYSNM<br>TLNETVRNVLYLANSTLSSNKNVAESGCKECEELEEKTFTEFLQSFI<br>RIVQMFINTSSNSLEVLFQGPTAAAAVSKGEELFTGVVPILVELDGDV<br>NGHKFSVSGEGEGDATYGKLTLKFICTTGKLPVPWPTLVTTLTYGV<br>QCFSRYPDHMKQHDFFKSAMPEGYVQERTIFFKDDGNYKTRAEVK<br>FEGDTLVNRIELKGIDFKEDGNILGHKLEYNYNSHNVYIMADKQKNGI<br>KVNFKIRHNIEDGSVQLADHYQQNTPIGDGPVLLPDNHYLSTQSALS<br>KDPNEKRDHMVLLEFVTAAGITLGMDELYK
IL-15Rα	MYSMQLASCVTLTLVLLVNSTTCPPPVSIEHADIRVKNYSVNSRERY<br>VCNSGFKRKAGTSTLIECVINKNTNVAHWTTPSLKCIRDPSLAHYSP<br>VPTVVTPKVTSQPESPSPSAKEPEAFSPKSDTAMTTETAIMPGSRLT<br>PSQTTSAGTTGTGSHKSSRAPSLAATMTLEPTASTSLRITEISPHSS<br>KMTK
IL-15Rα-GFP	MYSMQLASCVTLTLVLLVNSTTCPPPVSIEHADIRVKNYSVNSRERY<br>VCNSGFKRKAGTSTLIECVINKNTNVAHWTTPSLKCIRDPSLAHYSP<br>VPTVVTPKVTSQPESPSPSAKEPEAFSPKSDTAMTTETAIMPGSRLT<br>PSQTTSAGTTGTGSHKSSRAPSLAATMTLEPTASTSLRITEISPHSS<br>KMTKSNSLEVLFQGPTAAAAVSKGEELFTGVVPILVELDGDVNGHK<br>FSVSGEGEGDATYGKLTLKFICTTGKLPVPWPTLVTTLTYGVQCFSR<br>YPDHMKQHDFFKSAMPEGYVQERTIFFKDDGNYKTRAEVKFEGDT<br>LVNRIELKGIDFKEDGNILGHKLEYNYNSHNVYIMADKQKNGIKVNFK<br>IRHNIEDGSVQLADHYQQNTPIGDGPVLLPDNHYLSTQSALSKDPNE<br>KRDHMVLLEFVTAAGITLGMDELYK

Murine IL-2 signal sequence is written in red. Murine IL-15 and IL-15Rα are written in black. Linker and GFP sequences are written in blue and green, respectively.

Supplementary Figures

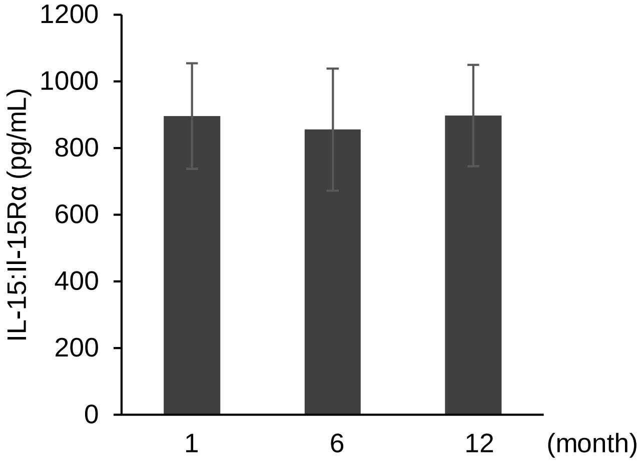

Figure S1. BacMam virus stability. BV stocks from the same batch were stored in the dark at 4 °C for the indicated number of months. B16F10 cells (5×10^5 cells/well) were infected at MOI of 10, using the BV stocks after storage for the indicated times. Culture supernatants were harvested after 24 h, and the level of IL-15:IL-15Rα was measured by ELISA.

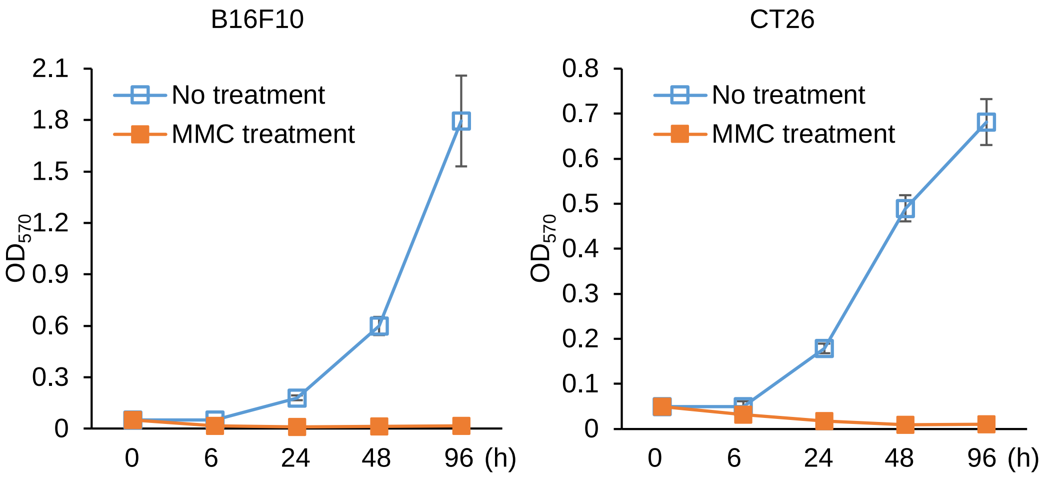

Figure S2. Proliferation assay of MMC treated tumor cells. B16F10-OVA and CT26-OVA were treated with MMC (50 μg/mL) for 1 h. The viability of tumor cells with or without MMC treatment was measured after 6, 24, 48 96 h using MTT assay.

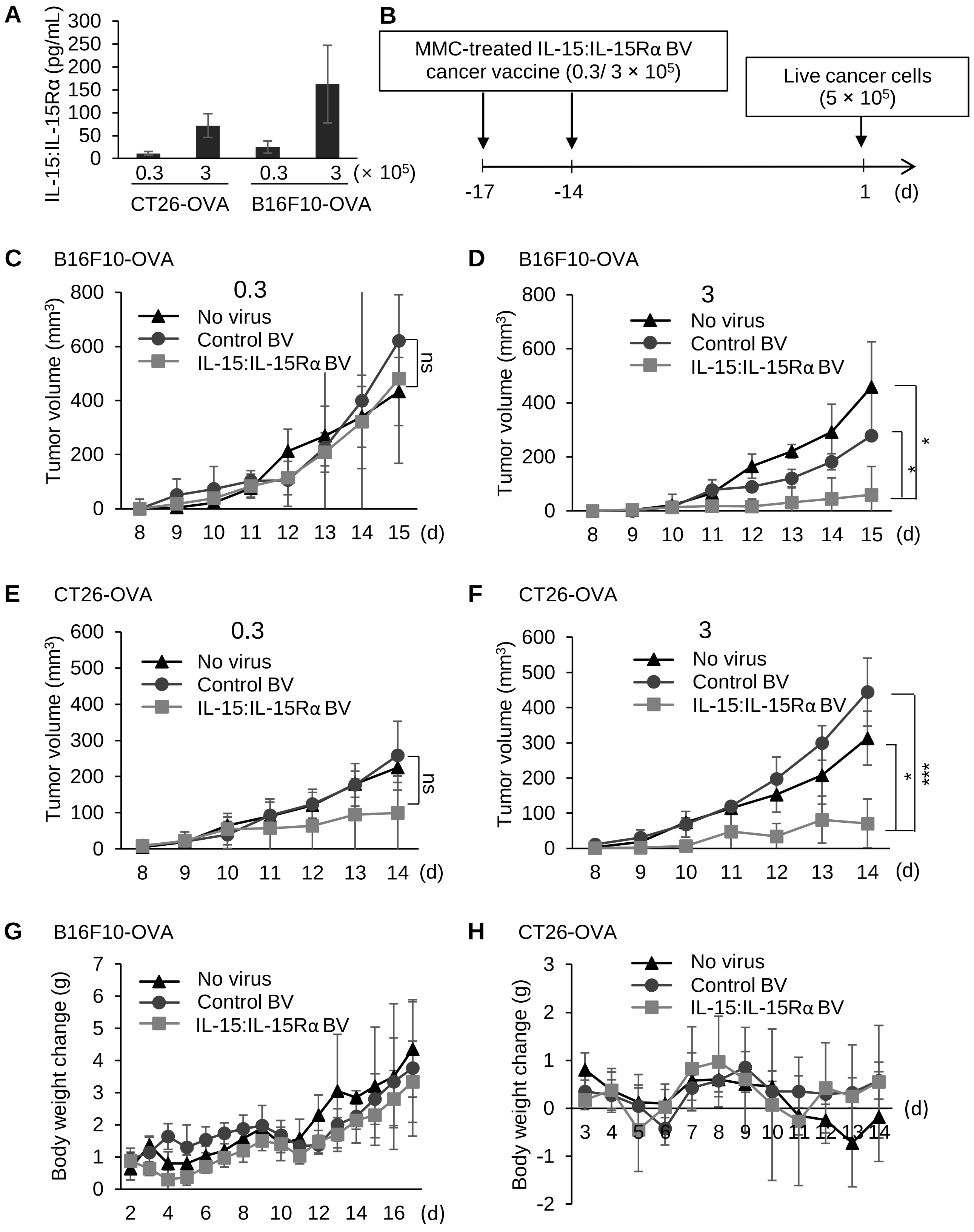

A
IL-15:IL-15Rα (pg/mL)
0.3 3 0.3 3 (× 10⁵)
CT26-OVA B16F10-OVA
B
MMC-treated IL-15:IL-15Rα BV cancer vaccine (0.3/ 3 × 10⁵)
Live cancer cells (5 × 10⁵)
-17 -14 1 (d)
C B16F10-OVA
0.3
Tumor volume (mm³)
No virus
Control BV
IL-15:IL-15Rα BV
ns
D B16F10-OVA
3
E CT26-OVA
0.3
F CT26-OVA
3
G B16F10-OVA
Body weight change (g)
H CT26-OVA

Figure S3. Determination of effective doses of BacMam-based IL-15:IL-15Rα cancer vaccine. (A) B16F10-OVA and CT26-OVA cancer cells were infected by a mixture of IL-15 BV and IL-15Rα BV at MOI of 8 and MOI of 16, respectively. The levels of IL-15:IL-15Rα complex, expressed by the infected cancer cells at 0.3 or 3 × 10^5 cell numbers, were measured by ELISA after 48 h. (B) The vaccination schedule of the dose-response experiments was shown. (C-F) The infected IL-15:IL-15Rα-B16F10-OVA and IL-15:IL-15Rα-CT26-OVA were harvested after 24 h and inactivated by MMC. Groups (n=3) of syngeneic C57/BL6J mice were subcutaneously immunized with the MMC-inactivated IL-15:IL-15Rα-B16F10 cells (C-D), or BALB/C mice were injected with IL-15:IL-15Rα-CT26 cells (E-F) at different cell numbers (0.3 or 3 × 10^5 cells/mouse) twice at an interval of 3 days. Two weeks after the last immunization, immunized mice were challenged with 5 × 10^5 live B16F10-OVA (C-D) or CT26-OVA (E-F) per mouse at the other flank. (G-H) The changes of body weights in mice of (D) and (F) experiments were shown. No virus, non-infected cells; control BV, control BV infected B16F10-OVA or CT26-OVA cells; IL-15:IL-15Rα BV, IL-15:IL-15Rα BV infected B16F10-OVA or CT26-OVA cells. *; $p < 0.05$, ***; $p < 0.001$.

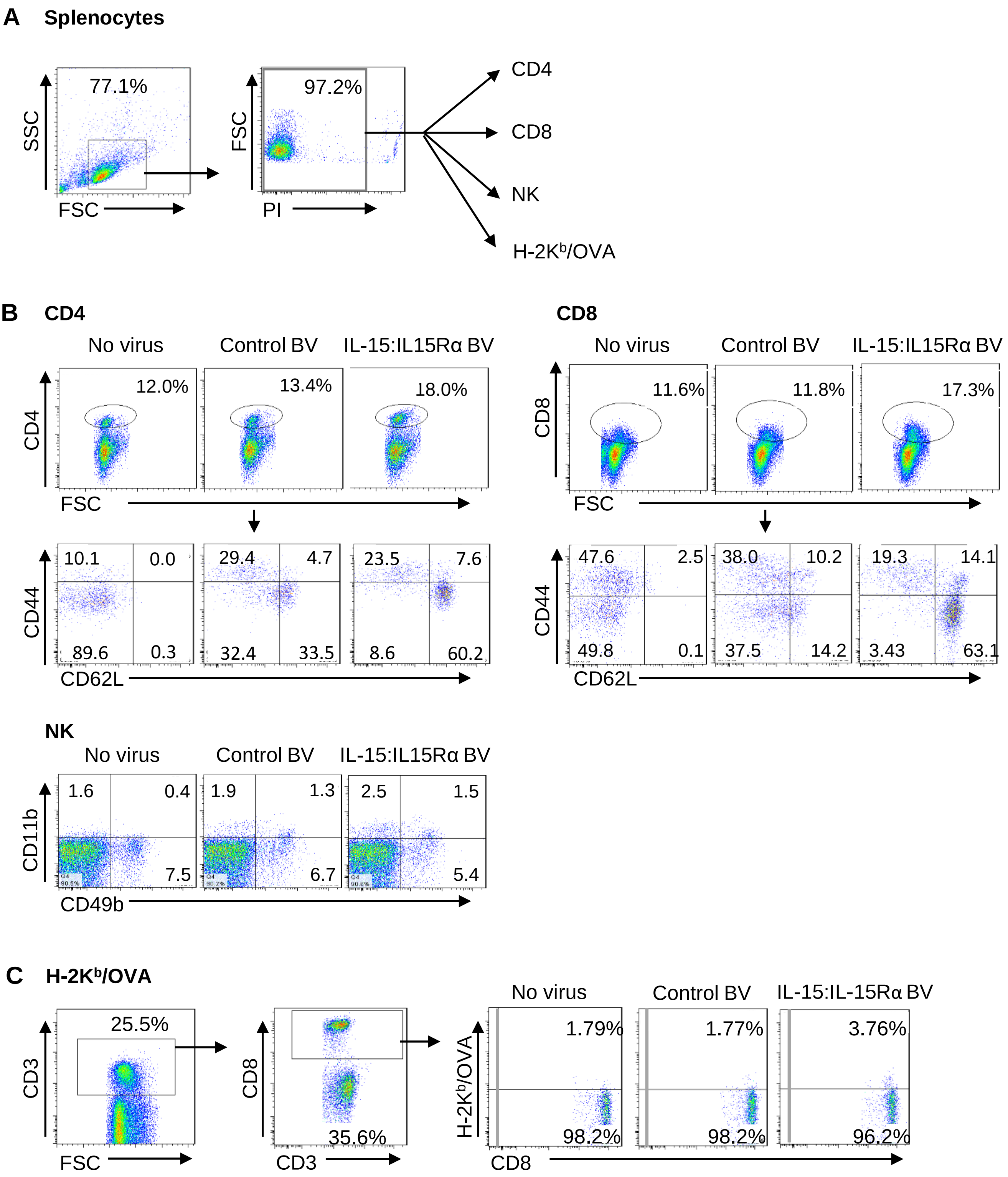

Figure S4. BacMam-based IL-15:IL-15Rα-B16F10-OVA vaccine increased tumor antigen-specific T cell and NK cell subsets in the spleen. Splenocytes were collected from mice vaccinated as described in Fig. 3A-C and stained to analyze for the percentage of effector memory ($CD44^{+}CD62L^{-}$) and central memory ($CD44^{+}CD62^{+}$) T cells, and NK subset (total NK; $CD49b^{+}$, effector NK; $CD49b^{+}CD11b^{+}$). FACS gating strategy used to analyze the splenocytes (A), FACS gating of CD4, CD8, and NK cells (B), FACS gating of OVA-specific cytotoxic T cells (H-$2K^{b}$/OVA (SIINFEKL)-$Tetramer^{+}CD3^{+}CD8^{+}$) (C).

PBMCs

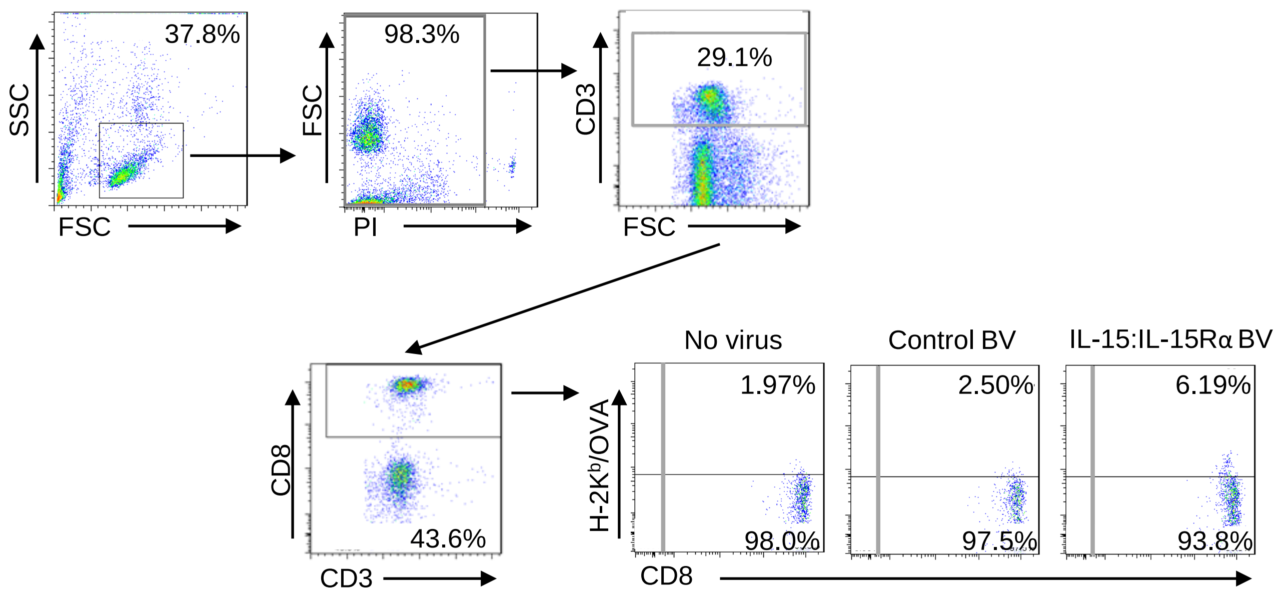

Figure S5. BacMam-based IL-15:IL-15Rα-B16F10-OVA vaccine increased OVA-specific CD8+ T cell subset in PBMC. PBMC were collected from mice vaccinated as described in Fig. 3A-C and stained to analyze for the percentage OVA-specific CD8+ T cells (H-2K^{b}/OVA (SIINFEKL)-Tetramer+CD3+CD8+).

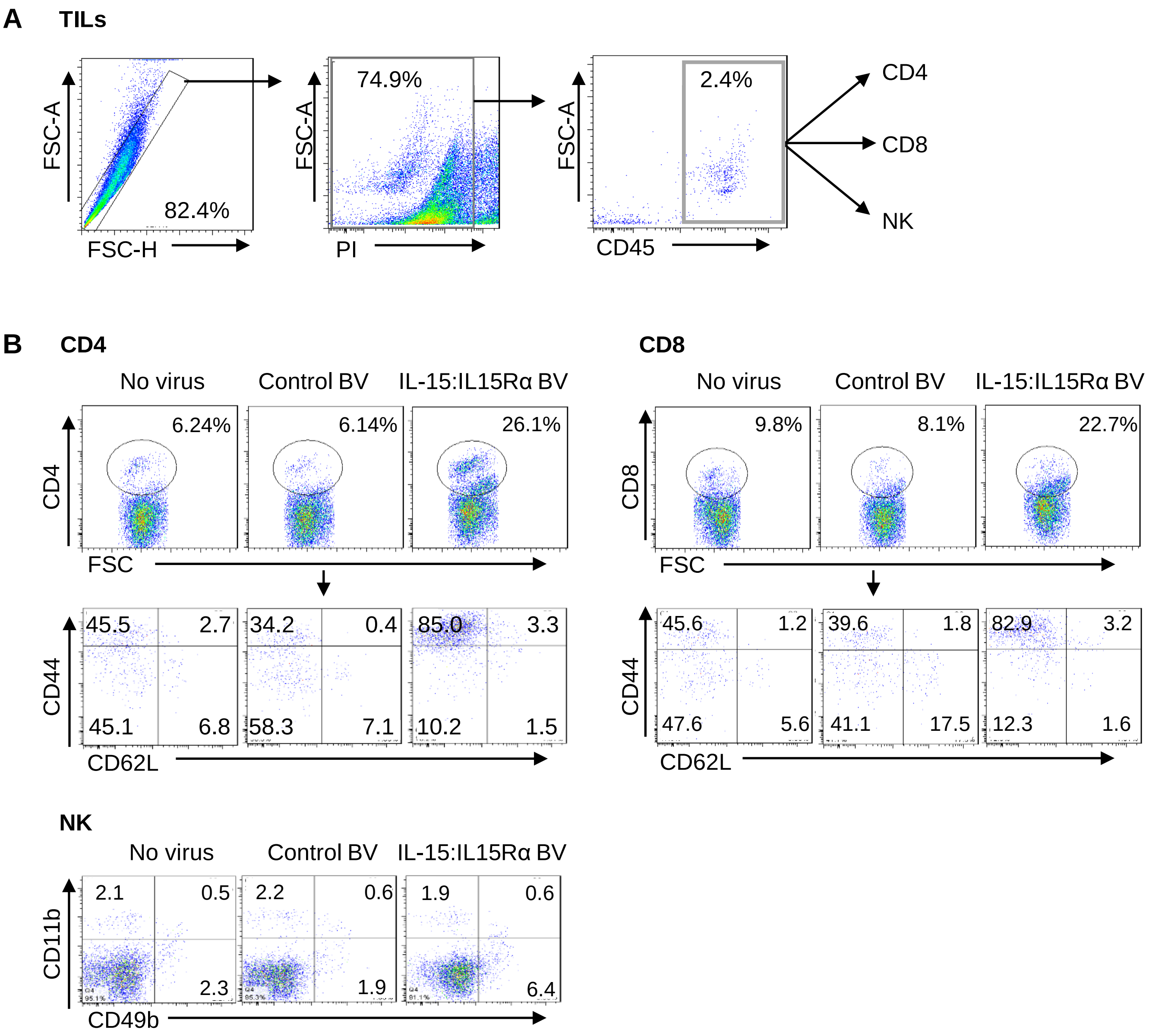

Figure S6. BacMam-based IL-15:IL-15Rα-B16F10-OVA vaccine increased T cell, and NK cell subsets in tumor infiltrated lymphocytes (TILs). TILs were collected from mice vaccinated as described in Fig. 3A-C and stained to analyze for the percentage of effector memory ($CD44^+CD62L^-$) and central memory ($CD44^+CD62^+$) T cells, and NK subset (total NK; $CD49b^+$, effector NK; $CD49b^+CD11b^+$). FACS gating strategy used to analyze the TILs (A), FACS gating of CD4, CD8, and NK cells (B).
